# Mechanism and Kinetics of HIV-1 Protease Activation

**DOI:** 10.3390/v16121826

**Published:** 2024-11-25

**Authors:** Caroline O. Tabler, John C. Tilton

**Affiliations:** Center for Proteomics and Bioinformatics, Department of Nutrition, School of Medicine, Case Western Reserve University, Cleveland, OH 44106, USA; cot3@case.edu

**Keywords:** HIV-1, protease, maturation, ribosomal frameshift, ESCRT, nanoscale flow cytometry

## Abstract

The HIV-1 protease is a critical enzyme for viral replication. Because protease activity is necessary to generate mature infectious virions, it is a primary target of antiretroviral treatment. Here, we provide an overview of the mechanisms regulating protease activation and the methods available to assess protease activity. Finally, we will highlight some of the key discoveries regarding the kinetics of protease activation from the last decade, including how the manipulation of activation kinetics may provide novel HIV-1 treatment strategies.

## 1. Introduction

**Figure 1:**
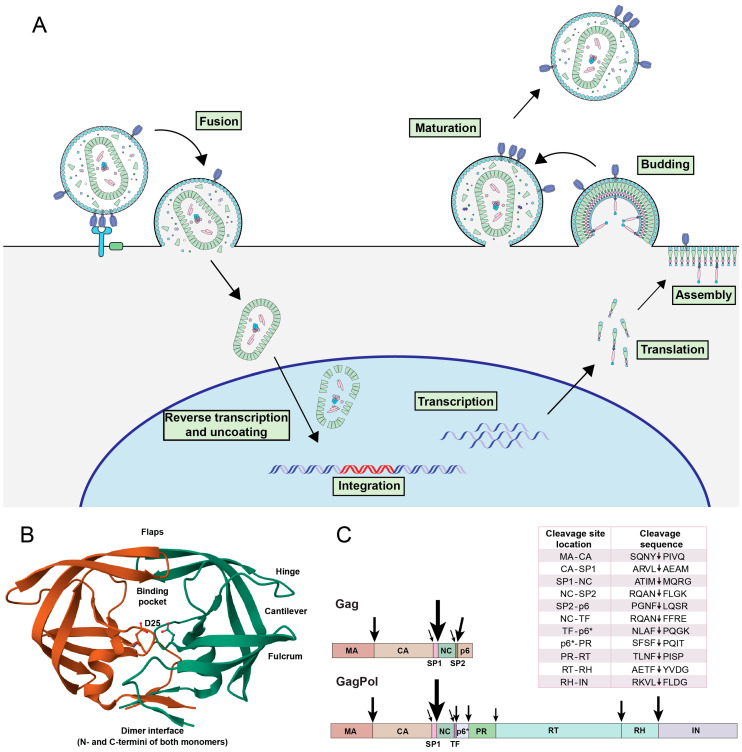
Role of HIV-1 protease during viral replication. (**A**) The HIV-1 replication cycle. The cycle begins with fusion, followed by nuclear trafficking of the capsid. The viral RNA genome is imported into the nucleus, where it is reverse transcribed, and the capsid shell uncoats (reviewed in [20]). The viral DNA is then integrated into the host chromosome, where it is transcribed and then translated. Finally, the virus assembles on the plasma membrane, where budding occurs. The final step is virus maturation mediated by the HIV-1 protease. (**B**) Ribbon structure of the mature HIV-1 protease dimer. Monomers are differentiated by color. Specific features, including the D25 catalytic residue and dimer interface, are noted. The structure was generated using the RCSB PDB (RCSB.org): PDB ID 4LL3 [21,22,23]. (**C**) The cleavage sites in Gag and GagPol are indicated by arrows, with the size of the arrow corresponding to the relative rate of processing. The HIV-1 B-clade consensus sequence of each cleavage site is listed in the table.

A critical step during HIV-1 replication is maturation, when the viral polyproteins Gag and GagPol are processed into functional subunits by the HIV-1 aspartic protease [1]. Gag and GagPol are translated within the host cell following infection and traffic to the plasma membrane with the help of a myristic acid modification at the Gag N-terminus (Figure 1A). There, they assemble with the viral fusogenic envelope (Env), accessory proteins, and cellular cofactors. Finally, the viral particle buds and is released from the cell. It is during viral budding that the HIV-1 protease, which is embedded in the GagPol polyprotein, forms dimers and becomes catalytically active [2].

Protease activity regulates both fusion and post-fusion events. The fusogenic Env protein has a relatively long ~150 amino acid cytoplasmic tail that is partially embedded in the viral membrane [3]. Interactions between the Env cytoplasmic tail and Gag ensure that Env is efficiently incorporated into viral particles [4,5,6]. However, the rigid lattice formed by unprocessed Gag stiffens the viral membrane, restricts the movement of Env, and prevents Env from obtaining a fusogenic conformation [7,8,9,10]. Once Gag is processed by protease, the softening of the viral membrane and the subsequent clustering of Env allows fusion to occur [7,8,9,10,11,12,13,14]. Protease is also required for the formation of a conical capsid shell within the virion that encapsulates the viral genome. The capsid protects the genome from innate viral sensors and helps to transport the genome into the nucleus following fusion [15,16,17,18]. Finally, the processing of the viral enzymes reverse transcriptase and integrase ensures their optimal function and promotes the efficient integration of viral DNA into the host cell chromosome [19].

The active protease dimer contains β-hairpin flaps that can adopt open, semi-open, and closed conformations with the help of flexible protease hinge domains (Figure 1B) [24,25]. The flaps open so that substrate can enter the binding pocket and then close around the substrate to position it next to the protease D25 residue that catalyzes peptide scission [25]. Protease can process a diverse array of substrates with varying cleavage sequences (Figure 1C). Protease binds to at least four amino acids on either side of the scission point, but processing efficiency is also influenced by more distal sequences that can interact with protease or alter the substrate structure [26,27]. Although substrates do not conform to a consensus sequence, there have been dozens of algorithms designed to identify substrates based on their sequence, structure, and other physiochemical properties [28,29,30,31]. Although this work is ongoing, a prevailing theory is that the protease recognizes substrates based on a common shape, with substrates forming a circular toroid shape on one side of the scissile point and a linear extended conformation on the other [32]. A more recent study by Potempa et al. found that the protease binding sites interacting with the substrate P2 and P2’ amino acids are bispecific, preferentially binding to either a polar or aliphatic amino acid depending on the nature of the P1’ amino acid [33]. This, in turn, regulates the rate of cleavage.

The viral Gag and GagPol polyproteins contain at least 11 distinct protease cleavage sites (Figure 1C). Gag processing is thought to occur first at the SP1-NC site, then at the MA-CA and SP2-p6 sites, and finally at the NC-SP2 and CA-SP1 sites [34]. GagPol processing was discovered by Pettit et al. to initiate intramolecularly at the SP1-NC site, followed by TF-p6* [35]. A subsequent study found processing next occurred at the RH-IN, MA-CA, RT-RH, p6*-PR, and finally PR-RT cleavage sites [36]. The completion of Gag and GagPol processing results in the formation of infectious mature particles.

In addition to its viral substrates, protease is capable of processing many cellular proteins, as reviewed in Centazzo et al. [37]. Over 120 cellular substrates have been identified in vitro, but it is unclear which of these processing events are important for infection [38]. It was recently reported that protease can process the antiviral restriction factor YTHDF3, an N6 methyladenosine reader that is packaged into virions and interferes with viral reverse transcription [39]. In addition, protease can promote cell death by processing cytosolic procaspase 8 and BCL-2, which are involved in apoptosis, and CARD8 (caspase recruitment domain-containing protein 8), which initiates pyroptosis [40,41,42,43].

Protease inhibitors are a key component of HIV-1 antiretroviral treatment. In 1995, saquinavir became the first protease inhibitor approved by the FDA, with nine additional protease inhibitors approved over the following decade. Protease inhibitors mimic protease substrates and bind to the protease dimer catalytic site. The newest protease inhibitors, darunavir and tipranavir, can also disrupt protease dimerization by binding to protease monomers [44]. First-generation protease inhibitors suffered from low bioavailability and required frequent dosing schedules, in part because protease inhibitors are metabolized by the hepatic enzyme CYP3A4. As a result, protease inhibitors are often combined with CYP3A4 inhibitors cobicistat or ritonavir to drastically increase their half-life [45].

A primary concern when using inhibitors is the development of drug-resistance mutations resulting in treatment failure. Resistance mutations can quickly develop when strict dosing regimens are not carefully maintained, causing drug concentrations to drop to suboptimal levels. Combining multiple inhibitors that target the viral enzymes protease, reverse transcriptase, and/or integrase reduces the likelihood of failure because resistance must develop to all drugs simultaneously. A major advantage of protease inhibitors is their relatively high barrier to resistance. In addition, newer protease inhibitors like darunavir interact more extensively with protease and select for a unique set of resistance mutations, meaning that darunavir can still be effective even after a virus is resistant to other protease inhibitors [46]. Current frontline therapy recommendations from the HHS Panel on Antiretroviral Guidelines for Adults and Adolescents, updated 12 September 2024, are to combine an oral, second-generation integrase strand transfer inhibitor (INSTI) with two nucleoside reverse transcriptase inhibitors in most people with HIV [47]. If resistance to INSTIs is suspected, a regimen combining boosted darunavir with two NRTIs can be utilized.

## 2. Mechanism of Protease Activation

**Figure 2:**
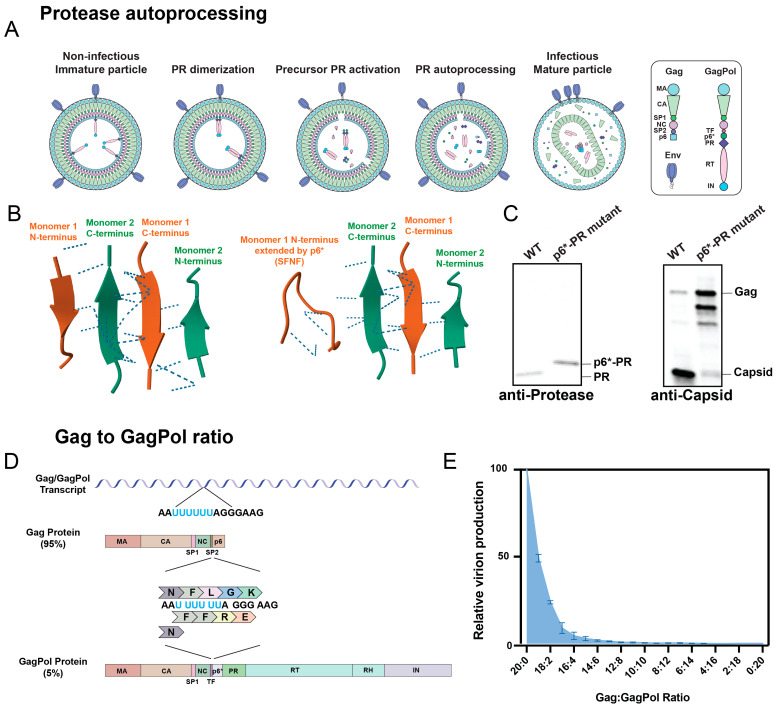

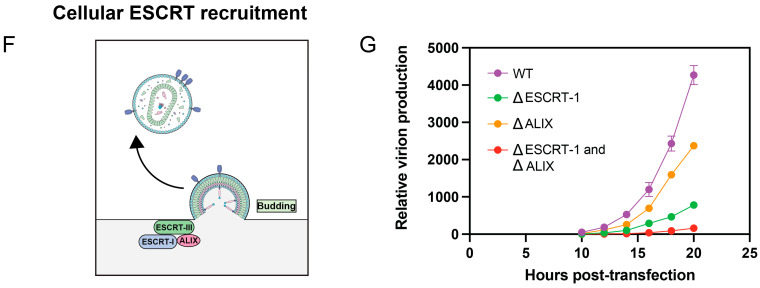
Regulation of protease activation kinetics. (**A**) Steps of HIV-1 protease maturation. In the immature particle, the protease embedded in GagPol must begin to dimerize for the precursor protease to activate, followed by N-terminal autoprocessing of p6* and mature protease activation. The end result is a fully mature, infectious viral particle. Republished with permission from Tabler et al. (2022) [57]. (**B**) Structure of protease dimer interface. When the N-termini of protease are fully processed, as in the first structure, a β-sheet is formed by the protease N- and C-termini that stabilizes dimerization. In the second structure, one of the monomers (Monomer 1, orange) has an extension on its N-terminus comprising the last four amino acids of p6* (SFNF). The addition of the unstructured p6* fragment alters hydrogen bonding, illustrated as dashed lines, within the dimer interface. This causes the β-sheet to unravel and compromises protease dimer stability. The structures were generated using the RCSB PDB (RCSB.org): PDB ID 4LL3 and 3TKW [21,22,23,52,58]. (**C**) The precursor protease has diminished processing capabilities. The p6*-PR cleavage site was mutated from SFSF-PQIT to SAAA-PQIT to prevent its processing in the NL4-3 HIV-1 clone. Compared to the wild-type (WT) virus, the mutant virus had a larger p6*-PR fusion protein, shown by an anti-protease Western blot, and it contained primarily unprocessed Gag, shown by an anti-capsid Western blot. Republished with permission from Tabler et al. (2022) [57]. (**D**) Mechanism of GagPol translation. The Gag/GagPol transcript has a slippery site containing six consecutive uracil residues. During translation, the ribosome translates the RNA as encoded 95% of the time, resulting in Gag production. The other 5% of the time, the ribosome undergoes-1 frameshifting that causes one of the uracil residues to be read twice, resulting in GagPol production. (**E**) Overexpression in GagPol results in loss of virion production. Gag and GagPol were cloned onto separate plasmids by mutating the frameshift site and were co-expressed at varying ratios in HEK293T cells. Gag:GagPol ratios higher than the typical 20:1 resulted in drastic reductions in viral production. Viruses were labeled with a fluorescent reporter and detected using nanoscale flow cytometry. Adapted with permission from Tabler et al. (2022) [57]. (**F**) The cellular ESCRT complex, including ESCRT-1, ESCRT-III, and ALIX, are recruited to the budding particle, where they assist in budding and membrane scission, allowing the viral particle to be released from the producer cell. (**G**) ESCRT recruitment is essential for HIV-1 production. The Gag p6 PTAP and YP motifs recruit the ESCRT-1 and ALIX proteins, respectively. These p6 motifs were mutated to LIRL and SR, respectively, to prevent the recruitment of ESCRT proteins, resulting in diminished production of viral particles due to premature protease activation. Viral particles were detected in the same manner as in (**E**). Adapted with permission from Tabler et al. (2022) [57].

Protease is embedded within the GagPol polyprotein, also referred to as GagProPol, when it first dimerizes and activates (Figure 2A). Active homodimers are stabilized primarily by a β-sheet formed by the first and last four amino acids of the protease monomers [2,48]. However, protease is initially translated with an unstructured peptide, p6*, attached to its N-terminus. The malleable p6* peptide prevents stable dimerization and limits protease function (Figure 2B) [49]. The protease dimer, including p6*, is referred to as the immature or precursor protease. The precursor protease only transiently dimerizes, with a dissociation rate 100-fold higher than that of mature protease [50,51,52]. Perhaps due to its unstable structure, protease inhibitors are significantly less effective at binding to and inhibiting the precursor protease [53,54,55]. Despite its impaired function, Western blot analysis shows that the precursor protease can inefficiently process many viral cleavage sites (Figure 2C), and our lab found evidence that most cleavage sites in Gag and GagPol are processed to a limited extent by the precursor protease using mass spectrometry [36,56]. Eventually, the precursor protease autoprocesses itself at the p6*-protease cleavage site, and the removal of p6* allows the mature protease to stably dimerize and process the remainder of Gag and GagPol [36,49,56].

Protease activation must be carefully timed to ensure efficient infection, and several mechanisms exist to prevent protease from activating prior to viral budding. This is because premature protease activation, which is when protease activates within the host cytosol prior to viral budding, inhibits particle production and causes the host cell to die, as will be discussed in Section 4. As just mentioned, p6* is essential to delaying protease activation, as the precursor protease containing p6* is much less active than the mature protease [59,60]. Activation is also partly regulated by protease concentration: the full-length HIV mRNAs encoding Gag and GagPol contain a ribosomal slip site in the Gag domain that regulates translation of the Pol domain, as Pol lacks an initiation codon of its own and is in a different reading frame than Gag (Figure 2D). The ribosome slips approximately 5% of the time, corresponding to a 20:1 ratio of Gag to GagPol expression [61]. Protease is within the Pol domain, and the limited translation of GagPol prevents protease from dimerizing and activating too soon (Figure 2E) [62]. In contrast, during viral assembly, the local concentration of GagPol is elevated, and interactions between other Pol proteins (reverse transcriptase, which dimerizes, and integrase, which tetramerizes) facilitate protease dimerization [63,64]. Normally, co-translational myristoylation of Gag facilitates its binding to the plasma membrane during viral assembly [65,66]. The expression of a mutant virus lacking myristoylation in a T-cell line caused intracellular protease activation to be inhibited, indicating that assembly at the membrane is important for GagPol to aggregate and initiate protease activation [67]. Finally, as the virus assembles at the cellular plasma membrane, the Gag protein p6 recruits cellular ESCRT (endosomal sorting complexes required for transport) proteins, including ALIX and ESCRT-I, which mediate efficient viral budding and membrane scission (Figure 2F) [68,69,70,71]. These cellular cofactors are required to accelerate the budding process, ensuring protease does not activate prematurely within the host cytosol (Figure 2G).

## 3. Methods to Analyze Protease Activation

### 3.1. Traditional Methods

**Figure 3:**
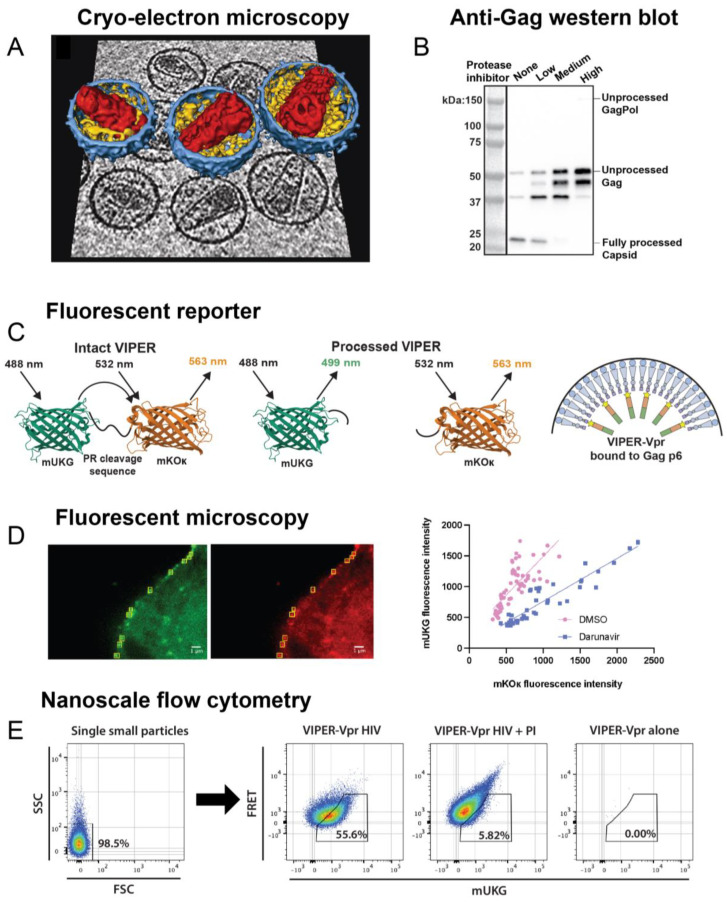

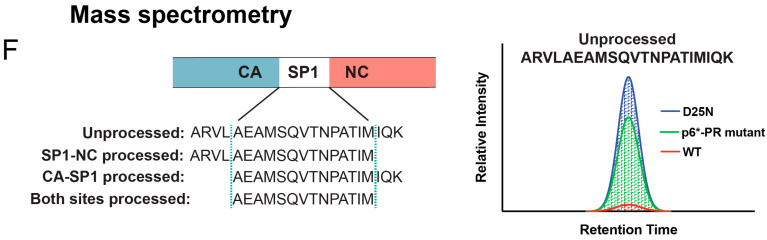
Methods to assess protease activity. (**A**) Cryo-electron microscopy was used to generate a tomogram that is overlayed with a 3D rendering of the three HIV-1 particles. The 3D rendering shows the viral membrane (blue), capsid (red), and density between the membrane and capsid (yellow). Republished with permission from Briggs et al. (2006) [79]. (**B**) An HIV-1 virus was treated with varying concentrations of the protease inhibitor amprenavir (0, 0.2, 1, or 5 μM) and analyzed with an anti-capsid antibody. As drug concentration increases, the fully processed capsid band disappears, and the relative amount of fully unprocessed Gag increases. (**C**) Schematic of fluorescent protease reporter. The example reporter, which we refer to as VIPER, consists of the fluorescent proteins mUKG (mUmikinoko-Green) and mKOκ (mKusabira-Orange-κ) separated by the protease cleavage sequence VSQNYPIVQN. When the reporter is intact, excitation of mUKG results in FRET and excitation of the nearby mKOκ protein. When protease processes the reporter, FRET is disrupted, and detectable mUKG fluorescence increases. The VIPER reporter can be linked to the viral protein Vpr, which non-covalently binds to the viral Gag p6 protein and is specifically incorporated into budding viral particles [80]. Representative fluorescent protein structures were generated using GFP from the RCSB PDB (RCSB.org): PDB ID 2QLE [21,81,82]. Republished under a Creative Commons CC BY 4.0 license from Tabler et al. (2024) [83]. (**D**) Representative microscopic image of the VIPER-Vpr reporter in 3C in viral puncta at the HEK293T cell membrane. HEK293T cells producing NL4-3 VIPER-Vpr were fixed and imaged using iSIM for mUKG and mKOκ fluorescence, with individual viral puncta segmented in yellow boxes. Cells treated with either DMSO or the protease inhibitor darunavir were compared for fluorescence intensity of both mUKG and mKOκ (61 puncta from 9 cells for the DMSO and 46 puncta from 8 cells for the darunavir). A simple linear regression was performed for each condition and plotted. The Spearman two-tailed correlation values were 0.8229 and 0.9225 for the DMSO-treated and darunavir-treated conditions, respectively. Republished with permission from Tabler et al. (2022) [57]. (**E**). HIV-1 particles produced with VIPER-Vpr shown in 3C were analyzed using nanoscale flow cytometry. The cytometer threshold was set based on mKOκ fluorescence, and the mKOκ channel voltage was adjusted such that only mKOκ-positive particles were recorded. Individual small particles were first gated using forward and side scatter, and then VIPER processing was gated based on increased mUKG fluorescence and decreased FRET. Wild-type HIV-1 resulted in 55.8% processing. The inclusion of a protease inhibitor (PI) like 5 µM darunavir prevented VIPER from being processed and was used as a negative control (6.28% processed events). Detection of VIPER-positive events in the absence of HIV-1, i.e., packaging of VIPER into extracellular vesicles, is minimal, with 0–1 events/second recorded (0.00% processed events). Republished under a Creative Commons CC BY 4.0 license from Tabler et al. (2024) [83]. (**F**) Simplified schematic of an LC-MS/MS assay to detect HIV-1 protease activity. Processing of the CA-SP1-NC peptide can result in the appearance of four potential peptides. When the peptides exit the chromatography column at a standard retention time, the peptides are detected and create peaks with an area relative to peptide concentration. The area of the peak is used to compare the concentration of identical peptides from different samples using label-free methods: for example, a wild-type (WT), catalytically inactive D25N protease mutant, and p6*-PR cleavage site mutant virus are compared.

There are many traditional methods available to study protease activity. Electron microscopy of virions shows a dense core following maturation, consisting of the fully formed capsid shell (Figure 3A). However, greater detail about processing specificity and kinetics can be obtained using other techniques, including protein gels and various reporter assays. 

Protein gels can detect the processing of most known protease substrates. The initial ground-breaking studies of protease activation kinetics from the Swanstrom lab involved pulse-chase analysis of Gag processing in the cytoplasm and membrane of infected cells [72,73]. Western blot analyses with anti-Gag antibodies, like against the Gag subunit capsid, are also commonly used to assess Gag processing (Figure 3B). Western blotting is capable of assessing protease activity within virions but requires many virions to be analyzed together in bulk, and it is not amenable to high-throughput analyses. It is also limited by the antibodies available for protein detection. These antibodies can be expensive, have unknown epitope specificities, or be prone to non-specific binding. When using a Western blot to perform quantitative analysis, variability in how well proteins transfer to the membrane and in how the densitometry analysis is performed can lead to inaccurate conclusions. Finally, some processing events are difficult to monitor by Western blot, even under the best conditions. For example, the processing of the CA-SP1 cleavage site decreases the apparent size of the capsid by less than 2 kDa, and this can be challenging to visualize.

In addition, numerous cell-based and cell-free assays have been developed to monitor protease activity. These assays typically involve a reporter substrate with an endogenous protease cleavage sequence (Figure 3C). Several types of reporters can be used to detect general protease activity, but many are fluorescence-based (reviewed in Neefjes et al.) [74]. For example, assays can include a peptide substrate that becomes fluorescent only after it is cleaved by recombinant protease. Other reporters use FRET (Förster or fluorescent resonance energy transfer) reporters, consisting of two fluorescent proteins linked by a protease cleavage sequence. If the reporter is intact, the energy from one excited fluorescent protein is immediately absorbed by the nearby second fluorescent protein. If the protease cleavage site is processed, the increased distance between the fluorescent proteins causes the FRET signal to decrease. The processing of reporters is typically detected using flow cytometry, microscopy, or a plate reader [75,76,77]. These assays are extremely useful because they can be highly sensitive, relatively inexpensive, and capable of high-throughput analyses of protease activity. Despite their benefits, most of these assays monitor recombinant protease function outside the context of an intact HIV-1 particle. It is increasingly clear that protease activation and function is regulated not only by protease itself but also by various elements within GagPol and the supramolecular assembly of the viral particle [67]. Therefore, assessing native protease activity within a virus is necessary to gain a complete understanding of how protease functions. Super-resolution microscopy may also be used to analyze protease activity in virions, for example, by visualizing the processing of a fluorescent reporter (Figure 3D) [78]. However, analysis is relatively time-consuming. Therefore, alternative assays, such as nanoscale flow cytometry and mass spectrometry, can be used to overcome many of these limitations.

### 3.2. Nanoscale Flow Cytometry

Unlike traditional assays, nanoscale flow cytometry allows individual and intact virions to be analyzed in a high-throughput manner. This technique has become increasingly feasible with the development of highly sensitive flow cytometers capable of assessing viral characteristics, including particle size and surface protein expression, as reviewed in Tabler et al. [84]. The principles of nanoscale flow cytometry are similar to those of cellular flow cytometry, with the primary exception being that viral particles are orders of magnitude smaller than cells: HIV-1 is approximately 100 nm in diameter, over 100 times smaller than a typical human cell. Although this makes analysis more complicated, specialized adaptations, including high-wattage and low-wavelength lasers, allow cytometers to detect small particles. However, size cannot be used to discriminate between viruses and extracellular vesicles, which are produced and released by cells at the same time as viral particles [85]. Luckily, virions can be specifically labeled with fluorescent markers, for example, through the incorporation of a fluorescent protein, genomic labeling, or staining with virus-specific fluorescent antibodies. This permits the exclusive identification of viral particles.

Our lab recently developed a nanoscale flow cytometry assay to study HIV-1 protease activity within viral particles (Figure 3E) [86]. Our assay makes use of a FRET-based reporter, VIPER-Vpr, described in Figure 3C. Protease activity is monitored based on the processing of the reporter, which results in increased mUKG fluorescence and decreased FRET. We confirmed that our assay accurately assessed protease activity in virions by screening known protease inhibitors that inhibited reporter processing [86]. In addition, viral concentration could be compared between samples based on the number of particles detected over the same amount of time by the cytometer [83].

### 3.3. Mass Spectrometry (MS)

One of the many applications of MS is to study proteolytic cleavage events. The HIV-1 protease can process several cellular proteins, many of which were discovered in the form of unexpected bands on protein gels. In contrast, MS can identify cellular substrates of protease in a proteome-wide and unbiased manner. For example, the lysate of a T-cell line was incubated with recombinant protease and screened for novel processing events using MS. A total of 123 cellular protease substrates were identified, only 2 of which had previously been reported to be processed by protease [38].

In addition to protein sequence, MS spectra can provide information about protein concentration (Figure 3F). This can be used to determine how efficiently a protein was processed. For protein quantification, both label-free and labeled methods are available. Label-free quantification involves comparing the intensity with which peptides are detected by a mass analyzer, which corresponds to the peptide abundance. This allows the relative concentration of identical peptides to be compared between different samples. Labeled methods are required to perform absolute peptide quantification [87]. This is typically done by spiking isotopically labeled peptides of known concentrations into the sample in order to serve as quantitative standards. Label-free MS quantification was used to assess how efficiently recombinant protease could process a substrate, with 202 compounds out of a pool of 24,032 found to inhibit protease with an IC_50_ less than 1 µM [75]. Similarly, a quantitative MS assay was previously used to monitor the rate of Gag processing in the presence of the maturation inhibitor bevirimat [88].

## 4. Discoveries Concerning Protease Activation Kinetics

### 4.1. Timing of Protease Activation

**Figure 4:**
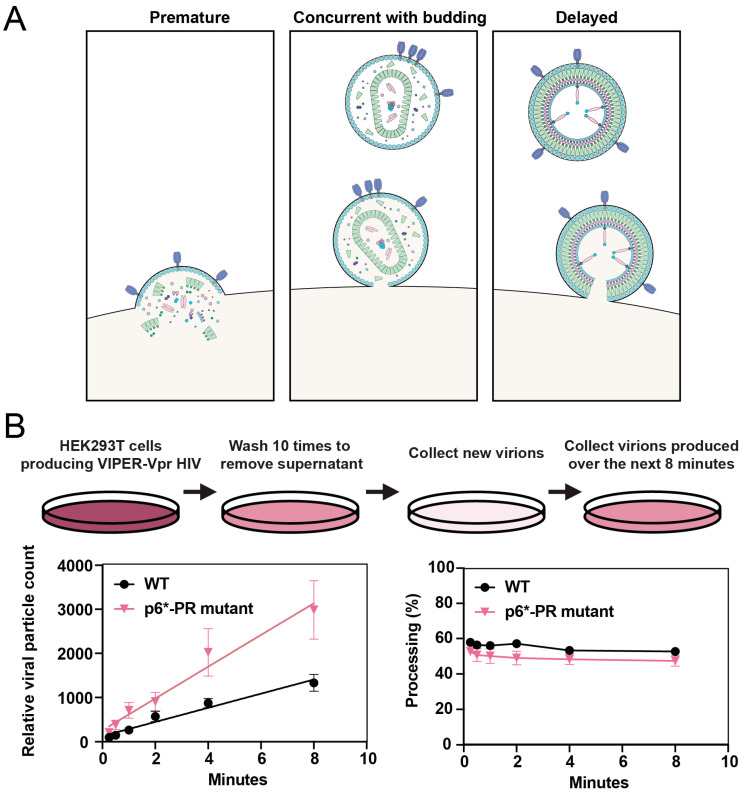
Kinetics of HIV-1 protease activation. (**A**) The three potential scenarios of when protease can be activated are illustrated. Premature protease activation occurs when the protease is activated within the cytosol of the infected cell, which results in the processing of essential viral proteins before the virus is formed and inhibits viral budding. It is thought that protease must be activated either concurrent with budding or shortly after viral release from the cell membrane to ensure viral replication continues. (**B**) Schematic of a washing experiment to analyze newly produced virions using nanoscale flow cytometry. HEK293T cells were co-transfected with NL4-3 HIV-1 and VIPER-Vpr, washed to remove residual virus, and new virions were collected over the following 8 min. The color of the cellular supernatant is reflective of the relative concentration of viral particles at each step of the experiment, with a darker color being the most concentrated. Relative particle concentration and the processing of NL4-3 VIPER-Vpr, with either WT NL4-3 or with a p6*-PR cleavage site mutation, was monitored over 10 successive washes. Four biological replicates were analyzed. The bars indicate the mean ± SEM. Republished with permission from Tabler et al. (2022) [57].

Resolving the kinetics of protease activation has been challenging. There have been several contradictory studies suggesting that protease activation can occur anywhere from during viral assembly to hours after particle release, possibly even after the endosomal uptake of virus into the target cell (Figure 4A) [57,72,73,89,90]. Pulse-chase analysis of Gag processing within infected cells revealed partially and fully processed Gag subunits in the membrane fraction, leading to the conclusion that protease dimerization is initiated at the cellular membrane during the assembly and budding process [72,73]. However, Western blot analysis of the membrane fraction can be confounded by antiviral factors, such as CD317 (tetherin), that attach viruses to the membrane even after they have completed membrane scission [91]. Western blot analysis of protease activation is further complicated by the requirement for millions to tens of millions of viral particles for even low-nanogram analysis, making it challenging to accumulate sufficient material to perform a second-by-second analysis of protease activity. 

We found that the processing activity detected by the nanoscale flow cytometry assay described in Figure 3E was comparable to that detected by Western blot [86]. Using this assay, we were able to capture and examine nascent virions and found that the precursor and mature protease are active less than a minute after viral release (Figure 4B) [57]. Furthermore, by analyzing viruses still associated with the cell membrane using Western blot and instant structured illumination microscopy, we demonstrated that protease is active in cells prior to viral release (Figure 3F) [57]. Our results further support earlier studies performed by the Swanstrom lab, showing that protease activation coincides with viral budding [72].

### 4.2. Delaying Protease Activation

The precise timing of protease activation is critical for successful viral replication, and protease inhibitors are an essential component of HIV-1 treatment. Protease inhibitors mimic protease substrates and strongly bind to the protease dimer catalytic site. The newest protease inhibitors, darunavir and tipranavir, also have an affinity for protease monomers and can disrupt protease dimerization [44].

**Figure 5:**
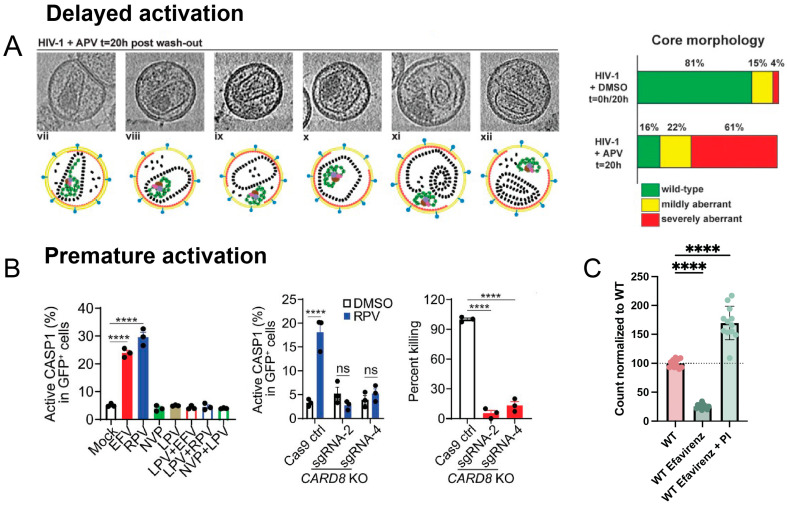
Studies altering the kinetics of protease activation. (**A**) The figure depicts computational slices through cryo-electron tomograms. Particles were analyzed following 20 h of incubation with the protease inhibitor amprenavir (APV) and inhibitor washout. Virions were classified according to their core morphology: wild-type, closed, ribonucleoprotein (RNP) inside (vii); mildly aberrant, closed, RNP inside (viii); mildly aberrant, closed, RNP outside (ix); severely aberrant, closed, RNP inside (x); severely aberrant, open, RNP outside (xi and xii). Compared to DMSO-treated virus, the protease inhibitor washout resulted in significantly more aberrant particles. More than 150 particles obtained from two independent preparations were analyzed per condition. Republished with permission from Mattei et al. (2014) [93]. (**B**) Non-nucleoside reverse transcriptase inhibitors (NNRTIs) induce caspase-1 activation and CARD8-mediated killing of infected primary CD4+ T cells. Activated primary CD4+ T cells were infected with HIV-1 NL4-3-Pol that expresses GFP following infection. On day 3 after infection, cells were treated with the NNRTIs efavirenz (EFV) and rilpivirine (RPV), the protease inhibitor lopinavir (LPV), or combinations thereof for 3 h before staining for active caspase-1 (CASP1) (left). The NNRTIs led to activation of caspase-1 that could be inhibited by a protease inhibitor. When caspase-1 could no longer activate due to CARD8 knockout (KO) (middle), there was reduced killing of infected CD4+ T cells 24 h after treatment with RPV, which was assessed based on the loss of GFP+ cells following treatment (right). New infections were blocked during treatment with the integrase inhibitor raltegravir and the fusion inhibitor T-20. *p* values were calculated using one-way ANOVA and Dunnett’s test (left, right) or using a two-way ANOVA and Tukey’s multiple-comparisons tests (middle). **** *p* < 0.0001. In each bar graph, *n* ≥ 3. Error bars indicate mean values with SEM. Data are representative of five or more independent experiments. Republished with permission from Wang et al. (2021) [41]. (**C**) Relative particle counts when cells producing VIPER-Vpr NL4-3 were treated with the reverse transcriptase inhibitor efavirenz or a combination of efavirenz and the protease inhibitor darunavir. Particle count was calculated as the number of events detected within 20 s of nanoscale flow cytometry analysis. Bars are the mean with the SEM. Adapted under a Creative Commons CC BY 4.0 license from Tabler et al. (2024) [83]. **** *p* < 0.0001. In each bar graph, *n* ≥ 3.

Partial inhibition or a mere delay in protease activation can severely reduce infectivity. These scenarios are likely to occur in vivo, where protease inhibitor concentrations vary throughout the body and over time. A modest impairment of protease activity caused by limiting drug concentrations resulted in outsized defects in infectivity and the formation of aberrant capsids [92]. Similarly, treatment with protease inhibitors followed by drug removal by washout allowed protease to regain function and process Gag (Figure 5A) [93]. However, despite near complete processing of Gag, viruses failed to regain infectivity due to both defective capsid formation, shown using electron microscopy, and incomplete maturation of the essential GagPol enzyme reverse transcriptase. These findings highlight the importance of controlled regulation and timing of protease activation.

### 4.3. Promoting Protease Activation

It is also possible for protease to activate too quickly while still in the host cytosol, which is referred to as premature protease activation. Premature activation causes Gag and GagPol to be processed before the virus has fully budded, resulting in the number and infectivity of viruses being impaired [94,95]. In addition, premature protease activation has been shown to activate several cell death pathways, notably by processing the cytosolic inflammasome sensor CARD8 that initiates pyroptosis [40,41]. This exciting finding suggests that drugs that induce premature protease activation can block both viral replication and selectively kill infected cells, making them of high interest in strategies to eliminate latent reservoirs in patients and cure infection [41,96,97].

In vitro studies have identified several ways to induce premature protease activation. For example, the overexpression of GagPol has been shown to cause premature protease activation, with even a modest increase in GagPol expression reducing particle release (Figure 2E) [98,99]. Molecules that modulate ribosomal frameshifting and increase the relative translation of GagPol have been investigated [100,101]. In addition, a delay in viral budding increases the chance that protease will intracellularly activate, and this may be accomplished by interfering with the recruitment of cellular ESCRT factors, which regulate the rate of budding (Figure 2G) [68,102]. Finally, the reverse transcriptase inhibitors efavirenz and rilpivirine have been shown to facilitate GagPol dimerization and, as a result, cause premature protease activation (Figure 5B) [41,96,97,103]. These drugs could potentially be a component of ‘kick and kill’ HIV-1 cure strategies, where latency reversal agents ‘kick’ latently infected cells so they produce viral proteins, and premature protease activators then induce pyroptosis and kill the infected cell. Although efavirenz and rilpivirine are capable of killing cells through premature protease activation in vitro, the concentration of drug required for cell death is too high to be effective in vivo, and the search for more potent drugs is ongoing. Merck has recently developed more targeted and potent premature protease activators to achieve cell killing at clinically relevant concentrations [104]. It was also recently discovered that inhibiting DPP9, a negative regulator of CARD8, can help to sensitize infected cells to protease-mediated pyroptosis [105].

Potential novel premature protease activators could include drugs that promote intracellular GagPol dimerization, increase GagPol translation by targeting ribosomal frameshifting, or delay budding by interfering with cellular ESCRT recruitment. High-throughput screens are often used to discover novel therapeutics, including HIV-1 protease inhibitors, and they may also be adapted to identify protease activators. Premature protease activators decrease viral budding, providing a simple measure with which to identify them. To distinguish between premature protease activators and drugs that inhibit viral budding through other mechanisms, drugs can also be tested in the presence and absence of a protease inhibitor. If premature protease activation is the cause, adding a protease inhibitor will abolish protease activity and restore particle production. We were able to observe this by comparing viral concentration using the VIPER-Vpr nanoscale flow cytometry system described in Figure 3E. Treatment with the reverse transcriptase inhibitor efavirenz, which is known to promote premature protease activation, caused virus production to decrease, but particle production was enhanced when co-treated with the protease inhibitor darunavir (Figure 5C). This provides evidence that premature protease activators can be identified using a high-throughput nanoscale flow cytometry screen, with later verification needed through their cell-killing action.

## 5. Conclusions

HIV-1 protease plays a central role during viral replication, regulating maturation, fusion, and the delivery and integration of the viral genome into the host chromosome. As a result, protease has been the subject of intensive study, and there has been a continuous series of new discoveries in the field.

The feat of defining HIV-1 protease activation kinetics has been ongoing for several decades, beginning with studies from the Swanstrom lab using pulse-chase analysis to discover processed Gag in the cytosol and membrane of infected cells [73]. Since then, new assays have been developed to provide greater insight into activation kinetics, including numerous protease reporter assays and nanoscale flow cytometry, which allows precursor and mature protease activity to be detected within seconds of viral formation. The recent finding that increasing the rate of protease activation kinetics simultaneously inhibits viral budding and kills the infected cell has kicked off a flurry of activity focused on inducing premature protease activation. With this exciting new avenue to explore, the kinetics of protease activation may be at the center of novel treatments for HIV-1 infection.

## Data Availability

The original contributions presented in this study (Figure 3B) are included in the article in the form of a cropped Western blot generated in our lab. Figure 3C,E and Figure 5C appeared in Tabler et al. (2024) [83]. Figure 2A,C,E,G, Figure 3D and Figure 4B appeared in Tabler et al. (2022) [57]. Figure 3A appeared in Briggs et al. (2006) [79], Figure 5A appeared in Mattei et al. (2014) [93], and Figure 5B appeared in Wang et al. (2021) [41]. All figures were republished with permission. Further inquiries can be directed to the corresponding author.

## References

[1] Kohl N.E., Emini E.A., Schleif W.A., Davis L.J., Heimbach J.C., Dixon R.A., Scolnick E.M., Sigal I.S. (1988). Active Human Immunodeficiency Virus Protease Is Required for Viral Infectivity. Proc. Natl. Acad. Sci. USA.

[2] Wlodawer A., Miller M., Jaskólski M., Sathyanarayana B.K., Baldwin E., Weber I.T., Selk L.M., Clawson L., Schneider J., Kent S.B. (1989). Conserved Folding in Retroviral Proteases: Crystal Structure of a Synthetic HIV-1 Protease. Science.

[3] Murphy R.E., Samal A.B., Vlach J., Saad J.S. (2017). Solution Structure and Membrane Interaction of the Cytoplasmic Tail of HIV-1 Gp41 Protein. Structure.

[4] Dorfman T., Mammano F., Haseltine W.A., Göttlinger H.G. (1994). Role of the Matrix Protein in the Virion Association of the Human Immunodeficiency Virus Type 1 Envelope Glycoprotein. J. Virol..

[5] Freed E.O., Martin M.A. (1996). Domains of the Human Immunodeficiency Virus Type 1 Matrix and Gp41 Cytoplasmic Tail Required for Envelope Incorporation into Virions. J. Virol..

[6] Wyma D.J., Kotov A., Aiken C. (2000). Evidence for a Stable Interaction of Gp41 with Pr55(Gag) in Immature Human Immunodeficiency Virus Type 1 Particles. J. Virol..

[7] Kol N., Shi Y., Tsvitov M., Barlam D., Shneck R.Z., Kay M.S., Rousso I. (2007). A Stiffness Switch in Human Immunodeficiency Virus. Biophys. J..

[8] Chojnacki J., Staudt T., Glass B., Bingen P., Engelhardt J., Anders M., Schneider J., Müller B., Hell S.W., Kräusslich H.-G. (2012). Maturation-Dependent HIV-1 Surface Protein Redistribution Revealed by Fluorescence Nanoscopy. Science.

[9] Roy N.H., Chan J., Lambelé M., Thali M. (2013). Clustering and Mobility of HIV-1 Env at Viral Assembly Sites Predict Its Propensity to Induce Cell-Cell Fusion. J. Virol..

[10] Joyner A.S., Willis J.R., Crowe J.E., Aiken C. (2011). Maturation-Induced Cloaking of Neutralization Epitopes on HIV-1 Particles. PLoS Pathog..

[11] Wyma D.J., Jiang J., Shi J., Zhou J., Lineberger J.E., Miller M.D., Aiken C. (2004). Coupling of Human Immunodeficiency Virus Type 1 Fusion to Virion Maturation: A Novel Role of the Gp41 Cytoplasmic Tail. J. Virol..

[12] Chojnacki J., Waithe D., Carravilla P., Huarte N., Galiani S., Enderlein J., Eggeling C. (2017). Envelope Glycoprotein Mobility on HIV-1 Particles Depends on the Virus Maturation State. Nat. Commun..

[13] Murakami T., Ablan S., Freed E.O., Tanaka Y. (2004). Regulation of Human Immunodeficiency Virus Type 1 Env-Mediated Membrane Fusion by Viral Protease Activity. J. Virol..

[14] Jiang J., Aiken C. (2007). Maturation-Dependent Human Immunodeficiency Virus Type 1 Particle Fusion Requires a Carboxyl-Terminal Region of the Gp41 Cytoplasmic Tail. J. Virol..

[15] Lahaye X., Satoh T., Gentili M., Cerboni S., Conrad C., Hurbain I., El Marjou A., Lacabaratz C., Lelièvre J.-D., Manel N. (2013). The Capsids of HIV-1 and HIV-2 Determine Immune Detection of the Viral CDNA by the Innate Sensor CGAS in Dendritic Cells. Immunity.

[16] Rasaiyaah J., Tan C.P., Fletcher A.J., Price A.J., Blondeau C., Hilditch L., Jacques D.A., Selwood D.L., James L.C., Noursadeghi M. (2013). HIV-1 Evades Innate Immune Recognition through Specific Cofactor Recruitment. Nature.

[17] Achuthan V., Perreira J.M., Sowd G.A., Puray-Chavez M., McDougall W.M., Paulucci-Holthauzen A., Wu X., Fadel H.J., Poeschla E.M., Multani A.S. (2018). Capsid-CPSF6 Interaction Licenses Nuclear HIV-1 Trafficking to Sites of Viral DNA Integration. Cell Host Microbe.

[18] Francis A.C., Melikyan G.B. (2018). Single HIV-1 Imaging Reveals Progression of Infection through CA-Dependent Steps of Docking at the Nuclear Pore, Uncoating, and Nuclear Transport. Cell Host Microbe.

[19] Abram M.E., Parniak M.A. (2005). Virion Instability of Human Immunodeficiency Virus Type 1 Reverse Transcriptase (RT) Mutated in the Protease Cleavage Site between RT P51 and the RT RNase H Domain. J. Virol..

[20] Müller T.G., Zila V., Müller B., Kräusslich H.-G. (2022). Nuclear Capsid Uncoating and Reverse Transcription of HIV-1. Annu. Rev. Virol..

[21] Sehnal D., Bittrich S., Deshpande M., Svobodová R., Berka K., Bazgier V., Velankar S., Burley S.K., Koča J., Rose A.S. (2021). Mol* Viewer: Modern Web App for 3D Visualization and Analysis of Large Biomolecular Structures. Nucleic Acids Res..

[22] Kožíšek M., Lepšík M., Grantz Šašková K., Brynda J., Konvalinka J., Rezáčová P. (2014). Thermodynamic and Structural Analysis of HIV Protease Resistance to Darunavir-Analysis of Heavily Mutated Patient-Derived HIV-1 Proteases. FEBS J..

[23] Grantz Saskova K., Rezacova P., Brynda J., Kozisek M., Konvalinka J. Structure of Wild-Type HIV Protease in Complex with Darunavir 2014. PDB.

[24] Hornak V., Okur A., Rizzo R.C., Simmerling C. (2006). HIV-1 Protease Flaps Spontaneously Open and Reclose in Molecular Dynamics Simulations. Proc. Natl. Acad. Sci. USA.

[25] Deshmukh L., Tugarinov V., Louis J.M., Clore G.M. (2017). Binding Kinetics and Substrate Selectivity in HIV-1 Protease-Gag Interactions Probed at Atomic Resolution by Chemical Exchange NMR. Proc. Natl. Acad. Sci. USA.

[26] Laco G.S. (2015). HIV-1 Protease Substrate-Groove: Role in Substrate Recognition and Inhibitor Resistance. Biochimie.

[27] Lee S.-K., Potempa M., Kolli M., Özen A., Schiffer C.A., Swanstrom R. (2012). Context Surrounding Processing Sites Is Crucial in Determining Cleavage Rate of a Subset of Processing Sites in HIV-1 Gag and Gag-Pro-Pol Polyprotein Precursors by Viral Protease. J. Biol. Chem..

[28] Kontijevskis A., Wikberg J.E.S., Komorowski J. (2007). Computational Proteomics Analysis of HIV-1 Protease Interactome. Proteins.

[29] Singh O., Su E.C.-Y. (2016). Prediction of HIV-1 Protease Cleavage Site Using a Combination of Sequence, Structural, and Physicochemical Features. BMC Bioinform..

[30] Shen H.-B., Chou K.-C. (2008). HIVcleave: A Web-Server for Predicting Human Immunodeficiency Virus Protease Cleavage Sites in Proteins. Anal. Biochem..

[31] Samant N., Nachum G., Tsepal T., Bolon D.N.A. (2022). Sequence Dependencies and Biophysical Features Both Govern Cleavage of Diverse Cut-Sites by HIV Protease. Protein Sci..

[32] Prabu-Jeyabalan M., Nalivaika E., Schiffer C.A. (2002). Substrate Shape Determines Specificity of Recognition for HIV-1 Protease: Analysis of Crystal Structures of Six Substrate Complexes. Structure.

[33] Potempa M., Lee S.-K., Kurt Yilmaz N., Nalivaika E.A., Rogers A., Spielvogel E., Carter C.W., Schiffer C.A., Swanstrom R. (2018). HIV-1 Protease Uses Bi-Specific S2/S2’ Subsites to Optimize Cleavage of Two Classes of Target Sites. J. Mol. Biol..

[34] Pettit S.C., Lindquist J.N., Kaplan A.H., Swanstrom R. (2005). Processing Sites in the Human Immunodeficiency Virus Type 1 (HIV-1) Gag-Pro-Pol Precursor Are Cleaved by the Viral Protease at Different Rates. Retrovirology.

[35] Pettit S.C., Everitt L.E., Choudhury S., Dunn B.M., Kaplan A.H. (2004). Initial Cleavage of the Human Immunodeficiency Virus Type 1 GagPol Precursor by Its Activated Protease Occurs by an Intramolecular Mechanism. J. Virol..

[36] Pettit S.C., Clemente J.C., Jeung J.A., Dunn B.M., Kaplan A.H. (2005). Ordered Processing of the Human Immunodeficiency Virus Type 1 GagPol Precursor Is Influenced by the Context of the Embedded Viral Protease. J. Virol..

[37] Centazzo M., Manganaro L., Alvisi G. (2023). Cellular Targets of HIV-1 Protease: Just the Tip of the Iceberg?. Viruses.

[38] Impens F., Timmerman E., Staes A., Moens K., Ariën K.K., Verhasselt B., Vandekerckhove J., Gevaert K. (2012). A Catalogue of Putative HIV-1 Protease Host Cell Substrates. Biol. Chem..

[39] Jurczyszak D., Zhang W., Terry S.N., Kehrer T., Bermúdez González M.C., McGregor E., Mulder L.C.F., Eckwahl M.J., Pan T., Simon V. (2020). HIV Protease Cleaves the Antiviral M6A Reader Protein YTHDF3 in the Viral Particle. PLoS Pathog..

[40] Yang H., Nkeze J., Zhao R.Y. (2012). Effects of HIV-1 Protease on Cellular Functions and Their Potential Applications in Antiretroviral Therapy. Cell Biosci..

[41] Wang Q., Gao H., Clark K.M., Mugisha C.S., Davis K., Tang J.P., Harlan G.H., DeSelm C.J., Presti R.M., Kutluay S.B. (2021). CARD8 Is an Inflammasome Sensor for HIV-1 Protease Activity. Science.

[42] Strack P.R., Frey M.W., Rizzo C.J., Cordova B., George H.J., Meade R., Ho S.P., Corman J., Tritch R., Korant B.D. (1996). Apoptosis Mediated by HIV Protease Is Preceded by Cleavage of Bcl-2. Proc. Natl. Acad. Sci. USA.

[43] Nie Z., Bren G.D., Vlahakis S.R., Schimnich A.A., Brenchley J.M., Trushin S.A., Warren S., Schnepple D.J., Kovacs C.M., Loutfy M.R. (2007). Human Immunodeficiency Virus Type 1 Protease Cleaves Procaspase 8 in Vivo. J. Virol..

[44] Koh Y., Matsumi S., Das D., Amano M., Davis D.A., Li J., Leschenko S., Baldridge A., Shioda T., Yarchoan R. (2007). Potent Inhibition of HIV-1 Replication by Novel Non-Peptidyl Small Molecule Inhibitors of Protease Dimerization. J. Biol. Chem..

[45] Rock B.M., Hengel S.M., Rock D.A., Wienkers L.C., Kunze K.L. (2014). Characterization of Ritonavir-Mediated Inactivation of Cytochrome P450 3A4. Mol. Pharmacol..

[46] De Meyer S., Hill A., Picchio G., DeMasi R., De Paepe E., de Béthune M.-P. (2008). Influence of Baseline Protease Inhibitor Resistance on the Efficacy of Darunavir/Ritonavir or Lopinavir/Ritonavir in the TITAN Trial. J. Acquir. Immune Defic. Syndr..

[47] Guidelines for the Use of Antiretroviral Agents in Adults and Adolescents with HIVS. https://www.ncbi.nlm.nih.gov/books/NBK586306/.

[48] Todd M.J., Semo N., Freire E. (1998). The Structural Stability of the HIV-1 Protease. J. Mol. Biol..

[49] Tessmer U., Kräusslich H.G. (1998). Cleavage of Human Immunodeficiency Virus Type 1 Proteinase from the N-Terminally Adjacent P6* Protein Is Essential for Efficient Gag Polyprotein Processing and Viral Infectivity. J. Virol..

[50] Tang C., Louis J.M., Aniana A., Suh J.-Y., Clore G.M. (2008). Visualizing Transient Events in Amino-Terminal Autoprocessing of HIV-1 Protease. Nature.

[51] Louis J.M., Clore G.M., Gronenborn A.M. (1999). Autoprocessing of HIV-1 Protease Is Tightly Coupled to Protein Folding. Nat. Struct. Biol..

[52] Agniswamy J., Sayer J.M., Weber I.T., Louis J.M. (2012). Terminal Interface Conformations Modulate Dimer Stability Prior to Amino Terminal Autoprocessing of HIV-1 Protease. Biochemistry.

[53] Louis J.M., Aniana A., Weber I.T., Sayer J.M. (2011). Inhibition of Autoprocessing of Natural Variants and Multidrug Resistant Mutant Precursors of HIV-1 Protease by Clinical Inhibitors. Proc. Natl. Acad. Sci. USA.

[54] Humpolíčková J., Weber J., Starková J., Mašínová E., Günterová J., Flaisigová I., Konvalinka J., Majerová T. (2018). Inhibition of the Precursor and Mature Forms of HIV-1 Protease as a Tool for Drug Evaluation. Sci. Rep..

[55] Davis D.A., Soule E.E., Davidoff K.S., Daniels S.I., Naiman N.E., Yarchoan R. (2012). Activity of Human Immunodeficiency Virus Type 1 Protease Inhibitors against the Initial Autocleavage in Gag-Pol Polyprotein Processing. Antimicrob. Agents Chemother..

[56] Ludwig C., Leiherer A., Wagner R. (2008). Importance of Protease Cleavage Sites within and Flanking Human Immunodeficiency Virus Type 1 Transframe Protein P6* for Spatiotemporal Regulation of Protease Activation. J. Virol..

[57] Tabler C.O., Wegman S.J., Chen J., Shroff H., Alhusaini N., Tilton J.C. (2022). The HIV-1 Viral Protease Is Activated during Assembly and Budding Prior to Particle Release. J. Virol..

[58] Agniswamy J., Sayer J., Weber I., Louis J. Crystal Structure of HIV Protease Model Precursor/Darunavir Complex 2012.

[59] Partin K., Zybarth G., Ehrlich L., DeCrombrugghe M., Wimmer E., Carter C. (1991). Deletion of Sequences Upstream of the Proteinase Improves the Proteolytic Processing of Human Immunodeficiency Virus Type 1. Proc. Natl. Acad. Sci. USA.

[60] Yu F.-H., Chou T.-A., Liao W.-H., Huang K.-J., Wang C.-T. (2015). Gag-Pol Transframe Domain P6* Is Essential for HIV-1 Protease-Mediated Virus Maturation. PLoS ONE.

[61] Jacks T., Power M.D., Masiarz F.R., Luciw P.A., Barr P.J., Varmus H.E. (1988). Characterization of Ribosomal Frameshifting in HIV-1 Gag-Pol Expression. Nature.

[62] Lee S.-K., Potempa M., Swanstrom R. (2012). The Choreography of HIV-1 Proteolytic Processing and Virion Assembly. J. Biol. Chem..

[63] Takagi S., Momose F., Morikawa Y. (2017). FRET Analysis of HIV-1 Gag and GagPol Interactions. FEBS Open Bio.

[64] Hsieh S.-H., Yu F.-H., Huang K.-J., Wang C.-T. (2023). HIV-1 Reverse Transcriptase Stability Correlates with Gag Cleavage Efficiency: Reverse Transcriptase Interaction Implications for Modulating Protease Activation. J. Virol..

[65] Göttlinger H.G., Sodroski J.G., Haseltine W.A. (1989). Role of Capsid Precursor Processing and Myristoylation in Morphogenesis and Infectivity of Human Immunodeficiency Virus Type 1. Proc. Natl. Acad. Sci. USA.

[66] Bryant M., Ratner L. (1990). Myristoylation-Dependent Replication and Assembly of Human Immunodeficiency Virus 1. Proc. Natl. Acad. Sci. USA.

[67] Lee Y.M., Tian C.J., Yu X.F. (1998). A Bipartite Membrane-Binding Signal in the Human Immunodeficiency Virus Type 1 Matrix Protein Is Required for the Proteolytic Processing of Gag Precursors in a Cell Type-Dependent Manner. J. Virol..

[68] Bendjennat M., Saffarian S. (2016). The Race against Protease Activation Defines the Role of ESCRTs in HIV Budding. PLoS Pathog..

[69] Garrus J.E., von Schwedler U.K., Pornillos O.W., Morham S.G., Zavitz K.H., Wang H.E., Wettstein D.A., Stray K.M., Côté M., Rich R.L. (2001). Tsg101 and the Vacuolar Protein Sorting Pathway Are Essential for HIV-1 Budding. Cell.

[70] Strack B., Calistri A., Craig S., Popova E., Göttlinger H.G. (2003). AIP1/ALIX Is a Binding Partner for HIV-1 P6 and EIAV P9 Functioning in Virus Budding. Cell.

[71] Huang M., Orenstein J.M., Martin M.A., Freed E.O. (1995). P6Gag Is Required for Particle Production from Full-Length Human Immunodeficiency Virus Type 1 Molecular Clones Expressing Protease. J. Virol..

[72] Kaplan A.H., Manchester M., Swanstrom R. (1994). The Activity of the Protease of Human Immunodeficiency Virus Type 1 Is Initiated at the Membrane of Infected Cells before the Release of Viral Proteins and Is Required for Release to Occur with Maximum Efficiency. J. Virol..

[73] Kaplan A.H., Swanstrom R. (1991). Human Immunodeficiency Virus Type 1 Gag Proteins Are Processed in Two Cellular Compartments. Proc. Natl. Acad. Sci. USA.

[74] Neefjes J., Dantuma N.P. (2004). Fluorescent Probes for Proteolysis: Tools for Drug Discovery. Nat. Rev. Drug Discov..

[75] Meng J., Lai M.-T., Munshi V., Grobler J., McCauley J., Zuck P., Johnson E.N., Uebele V.N., Hermes J.D., Adam G.C. (2015). Screening of HIV-1 Protease Using a Combination of an Ultra-High-Throughput Fluorescent-Based Assay and RapidFire Mass Spectrometry. J. Biomol. Screen..

[76] Gaber R., Majerle A., Jerala R., Benčina M. (2013). Noninvasive High-Throughput Single-Cell Analysis of HIV Protease Activity Using Ratiometric Flow Cytometry. Sensors.

[77] Jin S., Ellis E., Veetil J.V., Yao H., Ye K. (2011). Visualization of Human Immunodeficiency Virus Protease Inhibition Using a Novel Förster Resonance Energy Transfer Molecular Probe. Biotechnol. Prog..

[78] Sood C., Francis A.C., Desai T.M., Melikyan G.B. (2017). An Improved Labeling Strategy Enables Automated Detection of Single-Virus Fusion and Assessment of HIV-1 Protease Activity in Single Virions. J. Biol. Chem..

[79] Briggs J.A.G., Grünewald K., Glass B., Förster F., Kräusslich H.-G., Fuller S.D. (2006). The Mechanism of HIV-1 Core Assembly: Insights from Three-Dimensional Reconstructions of Authentic Virions. Structure.

[80] Selig L., Pages J.C., Tanchou V., Prévéral S., Berlioz-Torrent C., Liu L.X., Erdtmann L., Darlix J., Benarous R., Benichou S. (1999). Interaction with the P6 Domain of the Gag Precursor Mediates Incorporation into Virions of Vpr and Vpx Proteins from Primate Lentiviruses. J. Virol..

[81] Shu X., Remington S.J. GFP/S205V Mutant 2008. PDB.

[82] Shu X., Leiderman P., Gepshtein R., Smith N.R., Kallio K., Huppert D., Remington S.J. (2007). An Alternative Excited-State Proton Transfer Pathway in Green Fluorescent Protein Variant S205V. Protein Sci..

[83] Tabler C.O., Wegman S.J., Alhusaini N., Lee N.F., Tilton J.C. (2024). Premature Activation of the HIV-1 Protease Is Influenced by Polymorphisms in the Hinge Region. Viruses.

[84] Tabler C.O., Tilton J.C. (2024). Analysis of Individual Viral Particles by Flow Virometry. Viruses.

[85] Vestad B., Llorente A., Neurauter A., Phuyal S., Kierulf B., Kierulf P., Skotland T., Sandvig K., Haug K.B.F., Øvstebø R. (2017). Size and Concentration Analyses of Extracellular Vesicles by Nanoparticle Tracking Analysis: A Variation Study. J. Extracell. Vesicles.

[86] Bonar M.M., Tabler C.O., Haqqani A.A., Lapointe L.E., Galiatsos J.A., Joussef-Piña S., Quiñones-Mateu M.E., Tilton J.C. (2020). Nanoscale Flow Cytometry Reveals Interpatient Variability in HIV Protease Activity That Correlates with Viral Infectivity and Identifies Drug-Resistant Viruses. Sci. Rep..

[87] Bondarenko P.V., Chelius D., Shaler T.A. (2002). Identification and Relative Quantitation of Protein Mixtures by Enzymatic Digestion Followed by Capillary Reversed-Phase Liquid Chromatography-Tandem Mass Spectrometry. Anal. Chem..

[88] Lin Z., Cantone J., Lu H., Nowicka-Sans B., Protack T., Yuan T., Yang H., Liu Z., Drexler D., Regueiro-Ren A. (2016). Mechanistic Studies and Modeling Reveal the Origin of Differential Inhibition of Gag Polymorphic Viruses by HIV-1 Maturation Inhibitors. PLoS Pathog..

[89] Schimer J., Pávová M., Anders M., Pachl P., Šácha P., Cígler P., Weber J., Majer P., Řezáčová P., Kräusslich H.-G. (2015). Triggering HIV Polyprotein Processing by Light Using Rapid Photodegradation of a Tight-Binding Protease Inhibitor. Nat. Commun..

[90] Dale B.M., McNerney G.P., Thompson D.L., Hubner W., de Los Reyes K., Chuang F.Y.S., Huser T., Chen B.K. (2011). Cell-to-Cell Transfer of HIV-1 via Virological Synapses Leads to Endosomal Virion Maturation That Activates Viral Membrane Fusion. Cell Host Microbe.

[91] Neil S.J.D., Zang T., Bieniasz P.D. (2008). Tetherin Inhibits Retrovirus Release and Is Antagonized by HIV-1 Vpu. Nature.

[92] Kaplan A.H., Zack J.A., Knigge M., Paul D.A., Kempf D.J., Norbeck D.W., Swanstrom R. (1993). Partial Inhibition of the Human Immunodeficiency Virus Type 1 Protease Results in Aberrant Virus Assembly and the Formation of Noninfectious Particles. J. Virol..

[93] Mattei S., Anders M., Konvalinka J., Kräusslich H.-G., Briggs J.A.G., Müller B. (2014). Induced Maturation of Human Immunodeficiency Virus. J. Virol..

[94] Arrigo S.J., Huffman K. (1995). Potent Inhibition of Human Immunodeficiency Virus Type 1 (HIV-1) Replication by Inducible Expression of HIV-1 PR Multimers. J. Virol..

[95] Kräusslich H.G. (1991). Human Immunodeficiency Virus Proteinase Dimer as Component of the Viral Polyprotein Prevents Particle Assembly and Viral Infectivity. Proc. Natl. Acad. Sci. USA.

[96] Trinité B., Zhang H., Levy D.N. (2019). NNRTI-Induced HIV-1 Protease-Mediated Cytotoxicity Induces Rapid Death of CD4 T Cells during Productive Infection and Latency Reversal. Retrovirology.

[97] Jochmans D., Anders M., Keuleers I., Smeulders L., Kräusslich H.-G., Kraus G., Müller B. (2010). Selective Killing of Human Immunodeficiency Virus Infected Cells by Non-Nucleoside Reverse Transcriptase Inhibitor-Induced Activation of HIV Protease. Retrovirology.

[98] Karacostas V., Wolffe E.J., Nagashima K., Gonda M.A., Moss B. (1993). Overexpression of the HIV-1 Gag-Pol Polyprotein Results in Intracellular Activation of HIV-1 Protease and Inhibition of Assembly and Budding of Virus-like Particles. Virology.

[99] Park J., Morrow C.D. (1991). Overexpression of the Gag-Pol Precursor from Human Immunodeficiency Virus Type 1 Proviral Genomes Results in Efficient Proteolytic Processing in the Absence of Virion Production. J. Virol..

[100] Anokhina V.S., McAnany J.D., Ciesla J.H., Hilimire T.A., Santoso N., Miao H., Miller B.L. (2019). Enhancing the Ligand Efficiency of Anti-HIV Compounds Targeting Frameshift-Stimulating RNA. Bioorg. Med. Chem..

[101] Brakier-Gingras L., Charbonneau J., Butcher S.E. (2012). Targeting Frameshifting in the Human Immunodeficiency Virus. Expert Opin. Ther. Targets.

[102] Rheinemann L., Downhour D.M., Bredbenner K., Mercenne G., Davenport K.A., Schmitt P.T., Necessary C.R., McCullough J., Schmitt A.P., Simon S.M. (2021). RetroCHMP3 Blocks Budding of Enveloped Viruses without Blocking Cytokinesis. Cell.

[103] Figueiredo A., Moore K.L., Mak J., Sluis-Cremer N., de Bethune M.-P., Tachedjian G. (2006). Potent Nonnucleoside Reverse Transcriptase Inhibitors Target HIV-1 Gag-Pol. PLoS Pathog..

[104] Balibar C.J., Klein D.J., Zamlynny B., Diamond T.L., Fang Z., Cheney C.A., Kristoff J., Lu M., Bukhtiyarova M., Ou Y. (2023). Potent Targeted Activator of Cell Kill Molecules Eliminate Cells Expressing HIV-1. Sci. Transl. Med..

[105] Clark K.M., Kim J.G., Wang Q., Gao H., Presti R.M., Shan L. (2023). Chemical Inhibition of DPP9 Sensitizes the CARD8 Inflammasome in HIV-1-Infected Cells. Nat. Chem. Biol..

